# A novel termination site in a case of Stüve–Wiedemann syndrome: case report and review of literature

**DOI:** 10.3389/fped.2024.1341841

**Published:** 2024-04-02

**Authors:** Deepali Bhalla, Sunil Sati, Donald Basel, Vijender Karody

**Affiliations:** ^1^ Medical College of Wisconsin, Milwaukee, WI, United States; ^2^ Department of Pediatrics, Medical College of Wisconsin, Milwaukee, WI, United States

**Keywords:** Stüve–Wiedemann syndrome, JAK/STAT pathway, leukemia inhibitory factor receptor, skeletal dysplasia, Schwartz–Jampel syndrome Type 2, neonatal respiratory distress, dysautonomia, neonatal mortality

## Abstract

Stüve–Wiedemann syndrome (SWS) is a rare autosomal recessive disorder that is characterized by bowing of long bones, dysautonomia, temperature dysregulation, swallowing and feeding difficulties, and frequent respiratory infections. Respiratory distress and hyperthermic events are the leading causes of early neonatal death, and most patients are not expected to survive past infancy. Here, we report on the survival of a 5-year-old male with SWS, discussing his case presentation, providing a brief clinical course, and discussing the outcome. This case adds to the literature surrounding rare instances of childhood survivors of SWS and raises awareness for this syndrome to facilitate an earlier recognition, intervention, and genetic counseling for the families, thereby improving understanding of this disease and the health outcomes for the children affected by this condition.

## 1 Introduction

Stüve–Wiedemann syndrome (SWS) is a rare autosomal recessive disorder associated with pathogenic variants in the leukemia inhibitory factor receptor gene (*LIFR*) on chromosome 5p13.1, and presents with a high rate of mortality in the early neonatal period (1, 2). The hallmark constellation of symptoms documented in individuals affected by this condition are bowing of long bones, joint restriction, and dysregulation of autonomic nervous system, such as swallowing and breathing difficulties, problems with temperature regulation, and paradoxical sweating, as well as respiratory distress (2–5). The most common cause of death in neonatal period are hyperthermic crises and respiratory distress (5–8). SWS patients are also susceptible to rare and fatal fungal infections, which contributes to morbidity and mortality in these patients (9).

## 2 Case description

A male infant was born via cesarean-section at 39 weeks gestation and had a birth weight of 2.94 kg. Apgar scores were 8 and 9 at 1 and 5 min, respectively. He was admitted to the neonatal intensive care unit (NICU) on continuous positive airway pressure (CPAP) for antenatal diagnosis of skeletal dysplasia and respiratory distress. Immediately after NICU admission, the patient had worsening respiratory distress and hypoxia, requiring intubation and needle aspiration of a left pneumothorax. He required administration of surfactant while on 100% FiO2 and was eventually diagnosed with Persistent Pulmonary Hypertension (PPHN) for which he needed ventilation support by High Frequency Oscillation (HFOV), vasopressors (Epinephrine) and stress dose corticosteroids. Given his skeletal dysplasia and PPHN at birth, genetics team was consulted. Exome sequencing identified homozygous deletion variant c.1646delG in leukemia inhibitory factor receptor (*LIFR*) transcript NM_002310 9 (chr5:g.38499640delC). This variant is a frameshift, resulting in a novel termination site 4 amino acids downstream (p.G549Efs*4) which is associated with loss of function of the *LIFR* transcript and confirming a diagnosis of Stüve–Wiedemann syndrome (SWS). Once SWS was confirmed, neurology was consulted due to the high risk of dysautonomia, hyperthermia, and neuropathy associated with this condition. Gabapentin was started in the 3rd week of life to regulate autonomic system and presumed neuropathic pain, with good clinical response. Our patient had one episode of possible dysautonomia at 2 months of age with a high temperature of 40°C and no signs of infections. As per neurology recommendations, bromocriptine, a dopamine receptor agonist, was trialed with some response. There were no further episodes of dysautonomia needing bromocriptine during the NICU stay. He was started on enteral feeds (all via nasogastric tube) on day of life (DOL) 6 and a Gastrostomy tube was placed at 3 months of age for poor progression of oral skills. Due to the ophthalmic abnormalities, such as poor blink reflex and decreased corneal sensation associated with SWS, ophthalmology was consulted and recommended corneal lubrication. He had a prolonged course in the NICU with non-invasive ventilation [CPAP, Neurally Adjusted Ventilatory Assist (NAVA), and HFNC] before he was weaned off to room air and went home at 10 weeks of age. The patient was re-admitted to the hospital 2 weeks after discharge with a possible aspiration event. He needed HFNC for a couple of days, along with steroids, aggressive pulmonary toilet, and diuretics.

**Figure 1:**
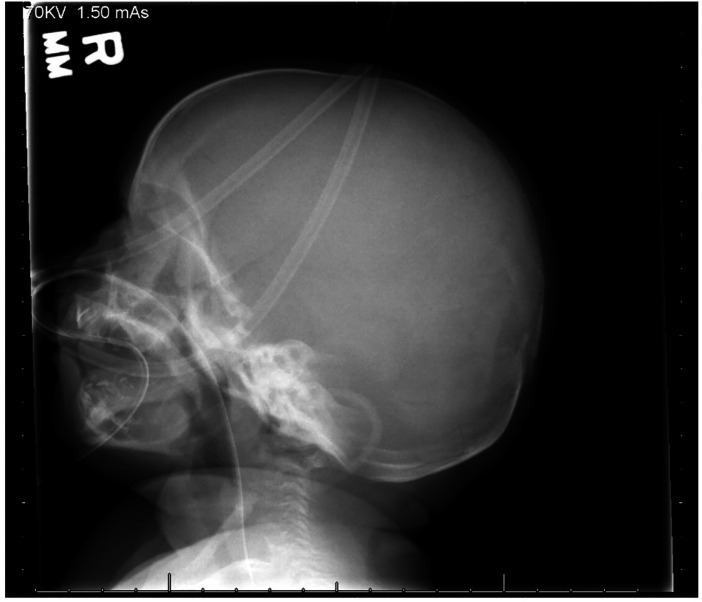
Apparent overriding of the lambdoid suture of the skull posteriorly is noted.

**Figure 2:**
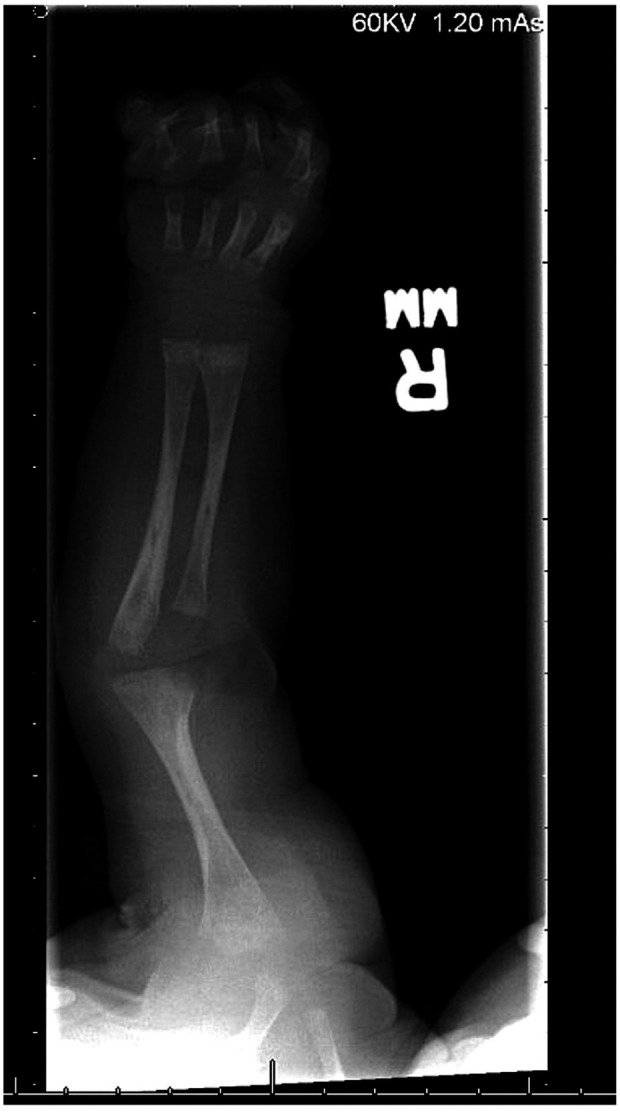
There is irregularity and bilateral widening of his proximal and distal humeral, radial, and ulnar metaphyses. Faint sclerotic metaphyseal bands within the metadiaphyses and metaphyses of the long bones is also observed. Mild cortical thickening is noted in the humeral diaphysis.

**Figure 3:**
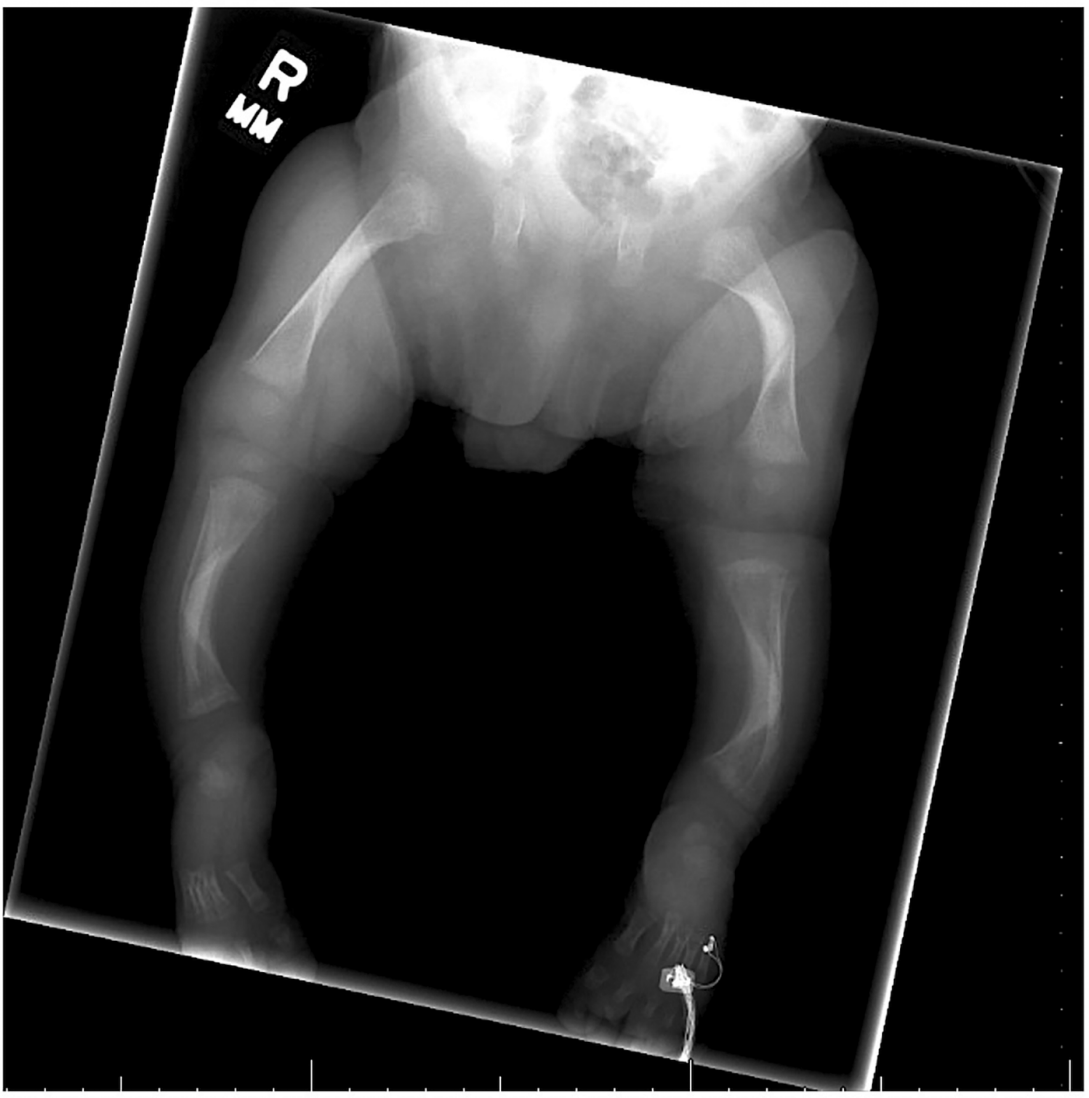
Radiologic images showing bowing of the bilateral femoral, tibial, and fibular diaphyses with smooth periosteal reaction along the bilateral tibial diaphyses. Diffuse mineralization of bones is present, indicating osteopenia. Bilateral cortical thickening in the middle diaphyses is also present in both the femur and tibia.

A skeletal survey performed at 5 weeks of age showed diffuse hypomineralization of bones, indicating osteopenia, and apparent overriding of the lambdoid suture of the skull posteriorly (Figure 1). Faint sclerotic metaphyseal bands within the metadiaphyses and metaphyses of the long bones of upper and lower extremities were present, and metaphyses of the long bones of the upper and lower extremities were noted to be broadened and irregular (Figure 2). Bowing of the bilateral femoral, tibial, and fibular diaphyses with smooth periosteal reaction and cortical thickening along the bilateral tibial diaphyses was also seen (Figure 3). A hip ultrasound was performed and was found to be normal.

As of writing this report, our patient is five years of age and retains the ability to ambulate independently despite having severe bilateral bowing of his lower legs. He attends physical therapy and is closely followed by the Orthopedic team for evaluation of his scoliosis, bilateral tibial bowing, coxa vara, and leg length discrepancy after bilateral femoral valgus osteotomy, which was performed at three years of age. He wears a Boston Thoracolumbrosacral Orthosis (TLSO) Brace to slow down spine curvature and delay spinal surgery. Plans for future corrective surgeries on his tibial bones are currently in discussion. He notably also developed bilateral club feet, camptodactyly, and enlargement of bilateral wrists. He is observed to have a prominent forehead, midface hypoplasia, dolichocephaly, low-set ears, absent Cupid's bow and U-shaped upper vermilion, and micrognathia. Due to his continued biting of the tongue during bruxism, our patient developed a traumatic fibroma on the lateral border of the tongue. With negative infection workup and confirmed SWS, the intermittent fevers in the first few weeks of life were thought to be secondary to dysautonomia and temperature dysregulation. Although he continues to have some temperature dysregulation, he has never experienced episodes of malignant hyperthermia in procedures that required administration of general anesthesia. He continues to take gabapentin for his neuropathic pain. He remains at a high risk for developing corneal abrasions and has developed exposure keratitis and corneal scaring due to his poor blink reflex; as well as punctate epithelial keratopathy, anisocoria, and decreased vision in both eyes. He continues to attend school and has preserved cognitive abilities.

## 3 Discussion

The first SWS case reported was found by Stüve and Wiedemann in 1971, where they described 2 sisters and a male cousin that shared phenotypical similarities of bowed legs, restricted joint movements, and other specific craniofacial and skeletal abnormalities; all of whom later developed respiratory distress leading to death in early neonatal period (10). Amongst the cases described in existing literature, most patients showed bowing of lower limb bones, camptodactyly, intrauterine growth restriction on second trimester and oligohydraminos in third trimester prenatal ultrasound (11). Bowing of lower limb bones such as tibia, and sparing fibula and upper limb bones, is one of the most prominent prenatal findings in SWS and reported in at least 46% of cases (8). There was initial confusion in differentiating SWS and Schwartz–Jampel Syndrome (SJS) Type 2 which seemed to share similar disease characteristics; but was later demonstrated to share the same molecular basis, thus establishing they were clinically and genetically analogous conditions (1).

In SWS, there is a loss of function pathogenic variant in *LIFR*, resulting in a truncated protein and inability of cytokines to bind appropriately (1, 4). Binding of the cytokine LIF to its receptor (LIFR) turns on JAK 1, which initiates tyrosine phosphorylation cascade activating JAK/STAT, MAP-kinase, and PI3-kinase signaling pathways (4, 6). These pathways play an important role in self renewal, differentiation, and survival of multiple tissues and organs. Since several tissues such as liver, kidney, bone, and central nervous system express LIFR, specific phenotypical characteristics expressed in SWS depend on which cytokines are involved in their signaling (6). It is theorized that absence of LIF and cardiotrophin-1 (CT-1) downstream signaling affects bone remodeling and informs the prominence of skeletal disruptions classically seen in SWS, whereas the impaired signaling of cardiotropin-like cytokine factor-1 (CLCF-1) contributes to the dysautonomia (4, 7). It is thought that pathogenic variants in *LIFR* gene alters motor neuron innervation, cholinergic differentiation, and survival of sympathetic neurons (1, 5, 12). The presence of dysautonomic symptoms in SWS places it in the same category of ciliary neurotrophic factor (CNTF) receptor related disorders, which includes Crisponi and cold-induced sweating syndrome. These syndromes share similar characteristics of SWS, including paradoxical sweating, feeding difficulties, and hyperthermic episodes (6, 12). CLCF-1 binds with cytokine receptor-like factor-1(CRLF-1), and competes with CNTF for the receptor complex which includes CNTFR, LIFR*,* and GP130 (12–14). Disturbance of this signaling caused by an abnormal *LIFR* gene product is the likely cause of dysautonomia and temperature dysregulation seen in SWS. Animal models used to study the effects of null *LIFR* gene variants in mice showed that those lacking CLCF-1 were found to have a reduced number of motor neurons in their facial nucleus and were unable to suckle appropriately, dying shortly after birth (12). This can explain the early feeding difficulties described in this syndrome, which was also documented in our patient. The dysphagia and facial muscle contractions observed in this syndrome are theorized to stem from absence in signaling between CLCF-1 and CRLF-1 (12). Furthermore, dysregulation of the LIFR-STAT3 signaling pathway is also seen in autosomal dominant hyperimmunoglobulin E syndrome, which shares several clinical characteristics with SWS such as increased risk of fungal infections, osteoporosis, and facial dysmorphia (9, 15).

There have been cases reported of patients demonstrating similar features of SWS without the *LIFR* gene pathogenic variant, and some have manifested incomplete phenotypes of SWS (such as dysautonomia without involvement of long bones) (8). Such instances prompt us to acknowledge the heterogenic nature of SWS cases and highlight the importance of modifying care for SWS patients on a case-by case basis. The characteristics of dysautonomia and temperature instability are the most common and prominent manifestations, especially in infancy (8). In a systematic review of 69 patients done by Warnier et al., mortality rate was found to be higher during the first 2 years of life (42% for ages less than 2 years as opposed to 10% for ages above 2 years of age) and was attributed mainly due to respiratory distress (71% of deaths) and dysautonomia (67%) (8). Mortality rates drastically decreased for the few that survived after 2 years of age. Due to extremely high rates of neonatal mortality, SWS was initially thought to be a lethal condition in the first year of life. Thus, cases reporting on patients surviving past 2 years such as this one are rare and are valuable to the literature and knowledge informing long term survival with SWS (8, 16, 17). Warnier et al. also reported in in their 2022 systematic review that Pulmonary arterial hypertension (PAH) during infancy was a poor prognosis factor (63% mortality rate). Skeletal deformities, while present in infancy, got progressively worse; requiring several orthopedic surgeries down the line (8, 16).

SWS exacts a high morbidity toll from individuals who survive long term. With age, there is progression of bowing in lone bones, severe spinal deformities such as scoliosis, osteoporosis, increasing spontaneous fractures, and joint movement restrictions, as seen in our patient. Despite skeletal abnormalities demanding several surgical procedures and specialized care, the interventions don't guarantee non-recurrence of deformities or improvement in mobility and walking (4, 5, 18). Still, early orthopedic interventions such as surgery and physiotherapy are crucial to prevent increasing the burden on patients' quality of life, and may require incorporating the use of bisphosphonates, vitamin D and calcium in the medical regimen to control recurrent fractures and accelerated osteoporosis (5, 7, 8, 19). Many long-term survivors require physical therapy and braces, as well as osteotomies to reduce limb deformities and surgical stabilization of scoliosis (8). Although motor and growth delay are present, cognitive impairment is not a feature of SWS (3, 4). As seen in our case, ocular manifestations are reported to become more prominent, with 45% experiencing corneal ulcers and 48% demonstrating a lack of corneal reflex in patients with SWS after 2 years of age (8). Neuropathy and reduced pain sensation in this syndrome carries a high risk of tongue biting injury, also seen in our patient, especially with the development of teeth (8). Dysautonomia continues to manifest throughout lifetime, and includes reduced pain sensation, temperature instability, paradoxical sweating, absent corneal reflexes, and smooth tongue (16). Given temperature dysregulation and hyperthermic episodes in SWS patients, concern has been previously expressed for their possible predisposition to malignant hyperthermic (MH) episodes with the administration of general anesthesia. There is a technique described using ketamine and propofol to induce a “dissociative anesthesia” in a 27-year-old patient with SWS to bypass any opportunity for MH to arise (20). However, their patient did not experience any problems with general anesthesia in her past surgical procedures, and there is other published literature with multiple cases of SWS patients undergoing surgical procedures under anesthesia without a reported episode of malignant hyperthermia (6, 8, 21, 22).

Currently, no definitive treatment exists for this condition. Management of SWS is supportive and directed towards manifested clinical symptoms (3, 6). Increased caution should be paid towards managing swallowing difficulties with nasogastric tube placement or gastrostomy especially during the first few years of life, as resulting pulmonary aspirations from it contributes to infections and respiratory distress (6, 8, 19). Our patient has home management plans for hyperthermia and respiratory exacerbations in place. His respiratory symptoms remain well managed at this time, and he is followed closely by Pulmonology. He has a detailed fever management plan from his pediatrician which includes acetaminophen, ibuprofen followed by bromocriptine if there is no response. Bromocriptine is a dopamine receptor agonist that has been used for treating hyperpyrexia in Neuroleptic Malignant Syndrome (NMS) and other hyperthermia of central origin, but to the best of our knowledge, its use in SWS has not been reported before (23–25). We also used gabapentin to treat pain and agitation in our patient. Gabapentin has been described in prior literature for treating neonatal irritability and pain, with reported improvement in pain scores and decreased need of neurosedative medications (26, 27). We use gabapentin in our unit to treat visceral pain and delirium with an aim of weaning opioids and other sedation medicines (28). Although we are the first ones to report the use of these two medications in SWS with some response, future studies and case series are needed before any recommendations can be made for their routine use in SWS.

Earlier diagnosis of SWS can reduce unnecessary and extensive laboratory workup, assisting the healthcare team in caring for the child and connecting families with the appropriate genetic counseling in a timely manner (5, 16). Consulting the expertise of pediatric radiology and genetics is vital in ensuring an accurate and narrow differential (3). Periods of respiratory distress and hyperthermia with seemingly no known cause should further raise suspicion for this diagnosis.

## 4 Conclusion

Here, we describe a novel termination site in a case of SWS who survived this rare disease past infancy. SWS is a clinical genetic syndrome with high morbidity and mortality in the first year of life. Early recognition and genetic diagnosis are helpful in a multidisciplinary team approach to the management. We also describe the use of gabapentin and bromocriptine for pain and hyperthermia management respectively in patients with SWS, which should be studied in future case reports and series.

## Data Availability

The original contributions presented in the study are included in the article/Supplementary Material, further inquiries can be directed to the corresponding author.
